# Robust quantum point contact via trench gate modulation

**DOI:** 10.1038/s41598-020-76790-z

**Published:** 2020-11-12

**Authors:** Dongsung T. Park, Seokyeong Lee, Uhjin Kim, Hyoungsoon Choi, Hyung Kook Choi

**Affiliations:** 1 Department of Physics, KAIST, Daejeon, 34141 Republic of Korea; 2 Department of Physics, Research Institute of Physics and Chemistry, Jeonbuk National University, Jeonju, 54896 Republic of Korea

**Keywords:** Electronic properties and materials, Quantum Hall, Electronic devices

## Abstract

Quantum point contacts (QPC) are a primary component in mesoscopic physics and have come to serve various purposes in modern quantum devices. However, fabricating a QPC that operates robustly under extreme conditions, such as high bias or magnetic fields, still remains an important challenge. As a solution, we have analyzed the trench-gated QPC (t-QPC) that has a central gate in addition to the split-gate structure used in conventional QPCs (c-QPC). From simulation and modelling, we predicted that the t-QPC has larger and more even subband spacings over a wider range of transmission when compared to the c-QPC. After an experimental verification, the two QPCs were investigated in the quantum Hall regimes as well. At high fields, the maximally available conductance was achievable in the t-QPC due to the local carrier density modulation by the trench gate. Furthermore, the t-QPC presented less anomalies in its DC bias dependence, indicating a possible suppression of impurity effects.

## Introduction

The quantum point contact (QPC) is the simplest, non-trivial quantum feature to be created on 2D electronic systems (2DES)^1,2^. By constricting the 2DES, it creates a set of local, quasi-1D subbands and allows for the spatial control of delocalized electrons^3,4^. Naturally, the structure serves as the elementary building block to many device architectures, such as quantum dots^5^, electron beam emitters^6,7^, and quantum Hall edge state beam splitters^8–10^. Despite its long history, the QPC still remains relevant as it not only provides a deep understanding of transport physics but also serves new purposes in modern quantum devices. For example, a clear characterization of 1D states is a benchmark for new methods or materials^11–13^, and even devices made in conventional settings can exhibit exotic states, such as zigzag Wigner crystals^14^, spin polarization^15^, and the controversial $0.7$ structure^16,17^. However, QPCs typically suffer from nonideal characteristics under extreme conditions, such as nonlinearities at high biases and transmissions or irregularities under magnetic fields, and the construction of a robust QPC still remains a crucial challenge.

QPCs in a 2DES are conventionally realized via split gate structures. The gates deplete part of the 2DES, and quasi-1D subbands form inside the gap due to the quantization in the constricted dimension^1–4^. A trench-gated QPC (t-QPC) includes an additional gate between the split gates; while the split gate forms a potential barrier, the trench gate can be used to further control the center of the constriction. The t-QPC has been used in the past to explore mesoscopic phenomena and unconventional 2DESs^18–21^, and several studies have sought to characterize the properties of the variant device geometry^22–25^. In particular, previous characterization studies emphasized the role of a trench gate in the enhancement of QPC subband spacings during split gate modulation. Here, we have characterized a t-QPC by modulating the trench gate rather than the split gate and compared it to the performance of a conventional QPC. The different behaviors have been modelled and corroborated through numerical simulations. From our analysis, we emphasize that trench gate modulation maintains a uniform large subband spacing even at higher numbers of conducting channels. Furthermore, the magnetoconductance have been investigated up to high magnetic fields, and the t-QPC was observed to be more robust against complications in the quantum Hall regime.

## Results and discussion

### Trench-gate QPC

**Figure 1:**
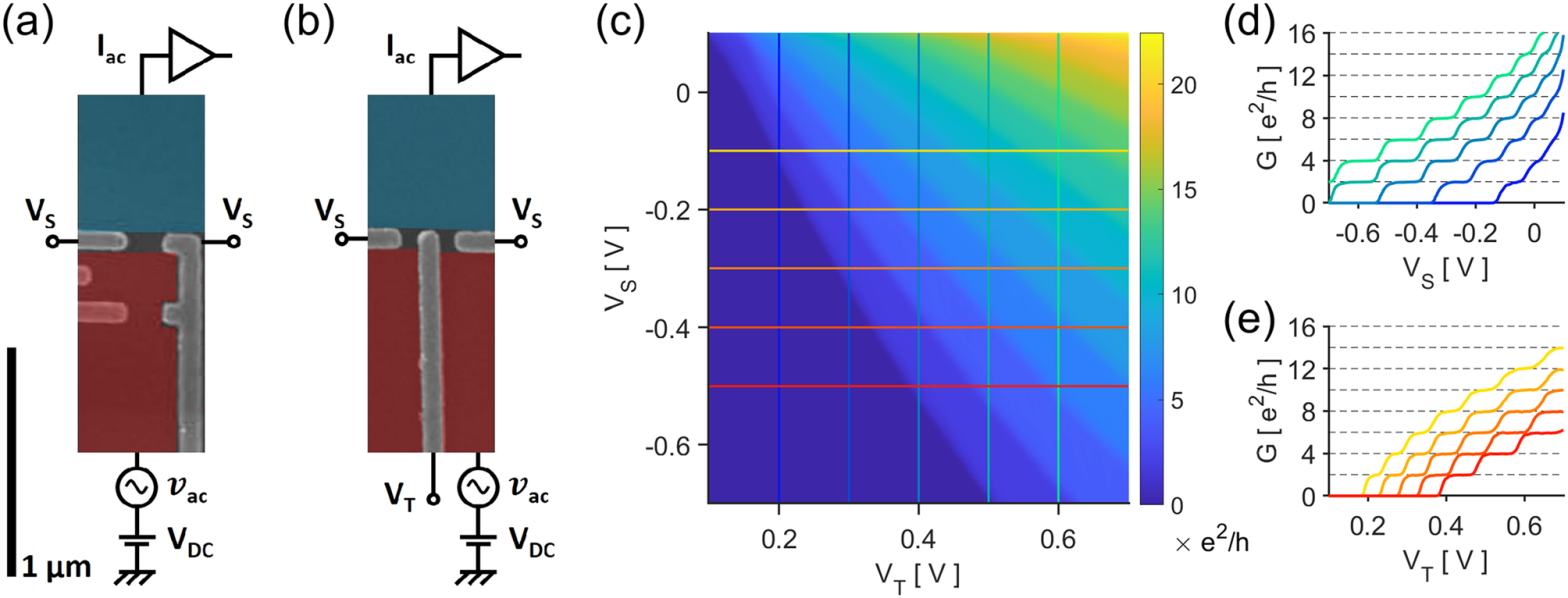
False colored SEM micrograph images of the nominal (**a**) c-QPC and (**b**) t-QPC devices used. The devices were measured using the 2-point probe scheme with a voltage source (red) and a current drain (blue). The c-QPC gates (grey) were imposed with a voltage ${\it{\text{V}}_{{\text{S}}}}$; the t-QPC split and trench gates with ${\it{\text{V}}_{{\text{S}}}}$ and ${\it{\text{V}}_{{\text{T}}}}$, respectively. (c) Raising ${\it{\text{V}}_{{\text{S}}}}$ or ${\it{\text{V}}_{{\text{T}}}}$ increases the t-QPC conductance. The conductance plateau is widened by either (d) raising ${\it{\text{V}}_{{\text{T}}}}$ or (e) lowering ${\it{\text{V}}_{{\text{S}}}}$.

A conventional QPC (c-QPC) consists of a single pair of split gates which are voltage-biased $V_{S}$ in order to deplete parts of the 2DES, Fig. 1a. This constricts the conducting path of the electrons. If the energy quantization in the constricted dimension is large enough, then the system can be locally described as multiple 1D subbands, each contributing to a quantized conductance of $2 \times e^{2} /h$ where $\times 2$ accounts for the spin degeneracy. In principle, a QPC controls the conductance only by modulating the constriction width. However, a trench gate placed between the split gates allows for the control of the potential at the center of the conducting path, Fig. 1b. The resulting conductance is the product of a competition between the two types of gates, Fig. 1c. By placing a positive voltage $V_{T}$ on the trench gate, the 1D subband energies are lowered, and previously unoccupied subbands may conduct if their subband minima fall below the Fermi level $E_{f}$.

The principal advantage of using a trench gate becomes apparent when we observe the energy spacing between the 1D subbands. The potential well in the constricted direction created by a negative $V_{S}$ is sharpened by a positive $V_{T}$^22^. In Fig. 1d, we see that a positive $V_{T}$ lengthens the conductance plateau in $V_{S}$—an indicator that the subband spacing has increased. Furthermore, the voltage at which the conduction vanishes has become lower, increasing the voltage range at which the device can be operated, i.e. the t-QPC has control over more energy-resolved 1D subbands. On the other hand, the t-QPC can also be operated by fixing the split gates $V_{S}$ and modulating the trench gate $V_{T}$ instead, Fig. 1e; the conductance is raised not by spatially widening the constriction but by bringing the subbands down to the Fermi level. In this sense, the wider subband spacing is more readily understood as the negative split gates squeezing the lateral potential well. In further discussion, we have focused on the effect of $V_{T}$ modulation with a fixed $V_{S} = - 0.45 {\text{V}}$.

### Modelling

**Figure 2:**
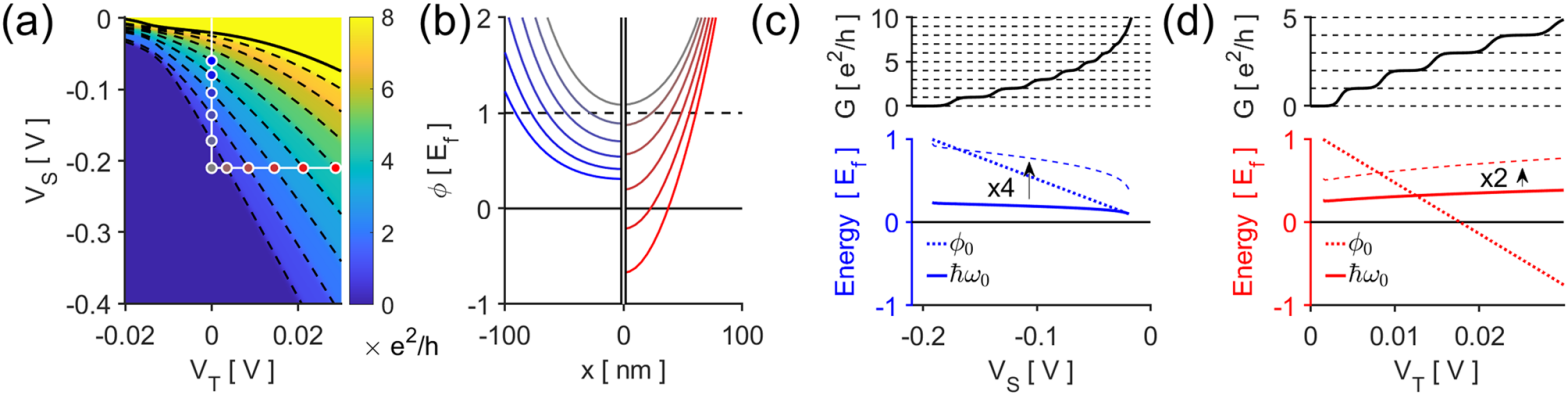
Simulation and modelling of QPCs. (**a**) The conductance of a simulated t-QPC, where ${\it{\text{V}}_{{\text{T}}}} = 0$ corresponds to a c-QPC. The constriction potential used for the colored points are plotted in (**b**); c-QPC in blue (left) and t-QPC in red (right). In order to raise the conductance, both QPCs increase in width and depth, but the t-QPC width is bounded by the presence of split gates. (**c**) The potential minimum and subband spacing of the c-QPC at various conductances obtained by approximating the potential as being harmonic. (**d**) A similar plot for the t-QPC.

In the semi-classical effective mass equation of motion^26^, the 2D Hamiltonian is given by

1 $$H_{2D} = \frac{{{\varvec{p}}^{2} }}{{2m^{*} }} + \phi \left( {\varvec{r}} \right)$$

where ${\varvec{p}} = \left( {p_{x} ,p_{y} } \right)$ is the linear momentum, ${\varvec{r}} = \left( {x,y} \right)$ the position, $m^{*}$ the effective mass, and $\phi \left( {\varvec{r}} \right)$ the electrostatic potential at ${\varvec{r}}$. Negatively biased split gates, $V_{S} < 0$, lead to a saddle-point potential which give rise to subbands continuous in the open direction while quantized in the constricted dimension^4^; the trench gate has the effect of modulating the potential at the saddle-point. A tight-binding calculation of the systems’ conductance shown in Fig. 2a, c.f. Methods for simulation details, clearly resembles experimental measurements in Fig. 1c.

The difference between QPCs is clearest in their confinement potentials. Figure 2b illustrates how the c-QPC (blue) and t-QPC (red) constricting potentials change as the conductance is raised. The constriction width $W$, defined as $\phi \left( {x = \pm W/2} \right) = E_{f}$, and the potential minimum $\phi_{0}$ are the two main factors. In c-QPCs, $W$ increases as $V_{S}$ becomes less negative—the gate potentials recede from the conducting region. Simultaneously, $\phi_{0}$ drops but is lower bounded by the original 2DES band minimum. In t-QPCs, on the other hand, $\phi_{0}$ can drop below said lower bound as $V_{T}$ becomes more positive. However, now $W$ is upper bounded due to the presence of split gates. Since the conductance of a tapered ballistic conductor is determined by the region of tightest constriction^27,28^, we can model the device by extending the lateral potential at the QPC center along the direction of the current. Here, we approximate the potential well as a simple harmonic potential:

2a $$\phi \left( {\varvec{r}} \right) = \frac{{m^{*} \omega_{0}^{2} }}{2}x^{2} + \phi_{0}$$

2b $$\omega_{0} = \frac{2}{W}\sqrt {\frac{{2\left( {E_{f} - \phi_{0} } \right)}}{{m^{*} }}}$$

2c $$\phi_{0} = - \alpha_{S} V_{S} - \alpha_{T} V_{T}$$

where $\omega_{0}$ represents the constriction strength and $- \alpha_{S,T} V_{S,T}$ account for changes in the potential minimum at the QPC center due to the gate potentials. The resulting 1D subband dispersion relations are given by

3 $$\varepsilon_{QPC} \left( {\sigma_{x} ,k_{y} } \right) = \frac{{\left( {\hbar k_{y} } \right)^{2} }}{{2m^{*} }} + \hbar \omega_{0} \left( {\sigma_{x} + \frac{1}{2}} \right) - \alpha_{s} V_{S} - \alpha_{T} V_{T}$$

where $\hbar k_{y}$ is the eigenvalue of $p_{y}$ and $\sigma_{x}$ is a nonnegative integer denoting the subband index. The subbands are spaced out by energy $\hbar \omega_{0}$, each with a conductivity $e^{2} /h$ in our spin-less model.

From the model, we see that the two types of QPCs exhibit different behaviors in their subband spacings. When raising the conductivity of c-QPC, both $\phi_{0}$ and $\hbar \omega_{0}$ decrease, Fig. 2c. Although $\phi_{0}$ affect the subband energies more, the simultaneous drop in $\hbar \omega_{0}$ is unavoidable; raising $V_{S}$ necessarily widens the QPC width, which weakens the constriction strength and therefore lowers the subband spacing, Eq. (2b). Furthermore, the maximum conductance of a c-QPC is limited by the $V_{S}$ range capable of depleting the 2DES. On the other hand, raising the t-QPC conductance lowers $\phi_{0}$ but increases $\hbar \omega_{0}$, Fig. 2d. Two points are significant. Firstly, $\phi_{0}$ is much more sensitive to changes in ${\text{V}}_{T}$ than it is to ${\text{V}}_{S}$, i.e. $\alpha_{T} \gg \alpha_{S}$. This is expected since the trench gate lies directly on top of the conducting region. Although not as pronounced as in the simulation, we see a similar trend in Fig. 1c. Second, $W$ seems to converge to the split gate positions and lowering $\phi_{0}$ can sharpen the confinement potential without affecting the constriction width. Thus, the t-QPC can drop more subbands under $E_{F}$ while keeping the split gates depleted by simply raising $V_{T}$. Therefore, it follows that the subband spacing can be raised by increasing the difference between gate voltages $V_{T}$ and $V_{S}$. Simply put, the ability of a t-QPC to modulate $\phi_{0}$ while maintaining a bounded $W$ allows the device to better control subbands with larger energy spacings.

#### Uniformity of subband spacings

**Figure 3:**
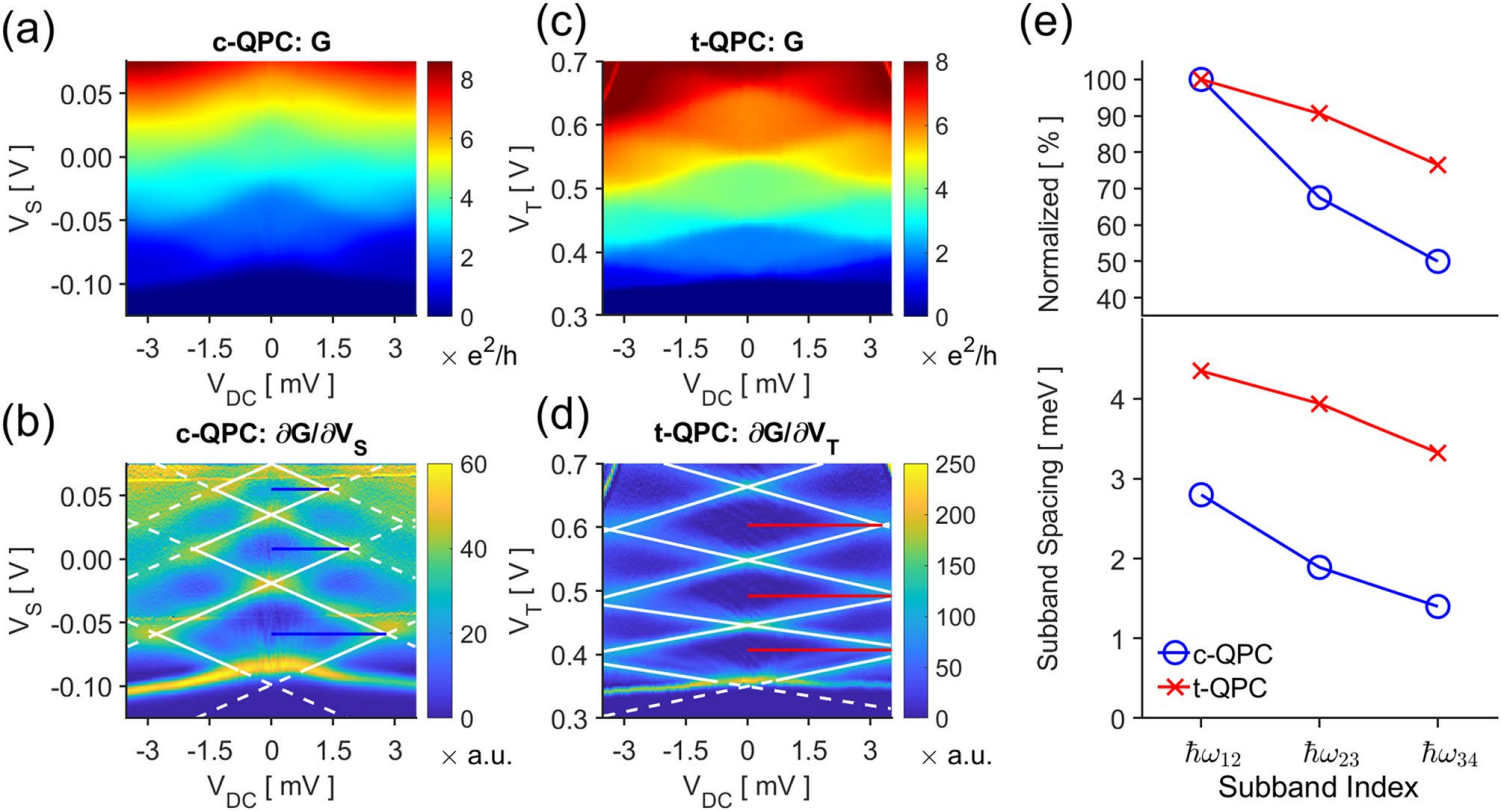
Source-drain biased measurements for the (**a**) c-QPC and (**c**) t-QPC. The differential conductances with respect to the gate voltages reveal where the subband minima coincide with ${\it{\text{E}}_{{\text{f}}}}$ or ${\it{\text{E}}_{{\text{f}}} - {\text{eV}}_{{{\text{DC}}}}}$; the voltage where (**b**) the c-QPC or (**d**) the t-QPC conductance changes have been indicated with white lines, outlining a series of rhombi along ${\it{\text{V}}_{{{\text{DC}}}}} = 0$. The horizontal halfwidths of rhombi (blue, red) is a direct measure of the subband spacings. (**e**) The t-QPC has a greater set of subband spacings than a c-QPC does, and the relative decrease of the spacings at higher conductances is more pronounced in c-QPCs.

The subband spacings can be directly measured by observing the dependence of the conductance plateaus on the source-drain bias $V_{DC}$. The conductance of a c-QPC for varying $V_{DC}$, Fig. 3a, indicates the mean number of subbands overlapping with the source and drain electrochemical potentials^29^. The transition between conductance plateaus become much more pronounced in $\partial G/\partial V_{S}$, Fig. 3b; the derivative is nonzero only when a subband minima is aligned either with $E_{F}$ (white lines, negative slope) or $E_{f} - eV_{DC}$ (white lines, positive slope). The horizontal halfwidths of the resulting rhombi correspond to the subband spacings, Fig. 3b blue lines. In our c-QPC, the energy difference between the first two subband minima $\hbar \omega_{12}$, i.e. the first subband spacing, is $2.80{\text{ meV}}$; but the second spacing drops to $1.89{\text{ meV}}$, and the third to $1.40{\text{ meV}}$—half of the first spacing.

Figure 3c,d show a similar plot for the t-QPC. We immediately observe a much larger set of subband spacings—$4.35{\text{ meV}}$, $3.94{\text{ meV}}$, and $3.33{\text{ meV}}$. Unlike the predictions from our model, we do not see an increase in the spacings. This can be attributed to the simple use of the pinned surface boundary condition when calculating the simulated potential^30^. The main effect unaccounted for is the 2DES electrons screening the gate potentials; a more positive trench gate increases the local carrier density which further broaden the potential well, resulting in the decrease in the subband spacings. Nevertheless, the normalized subband spacing clearly indicates that the trench gate still offers a more uniform set of subband spacings as our model suggests, Fig. 3e.

#### Magnetoelectric subband spacings

**Figure 4:**
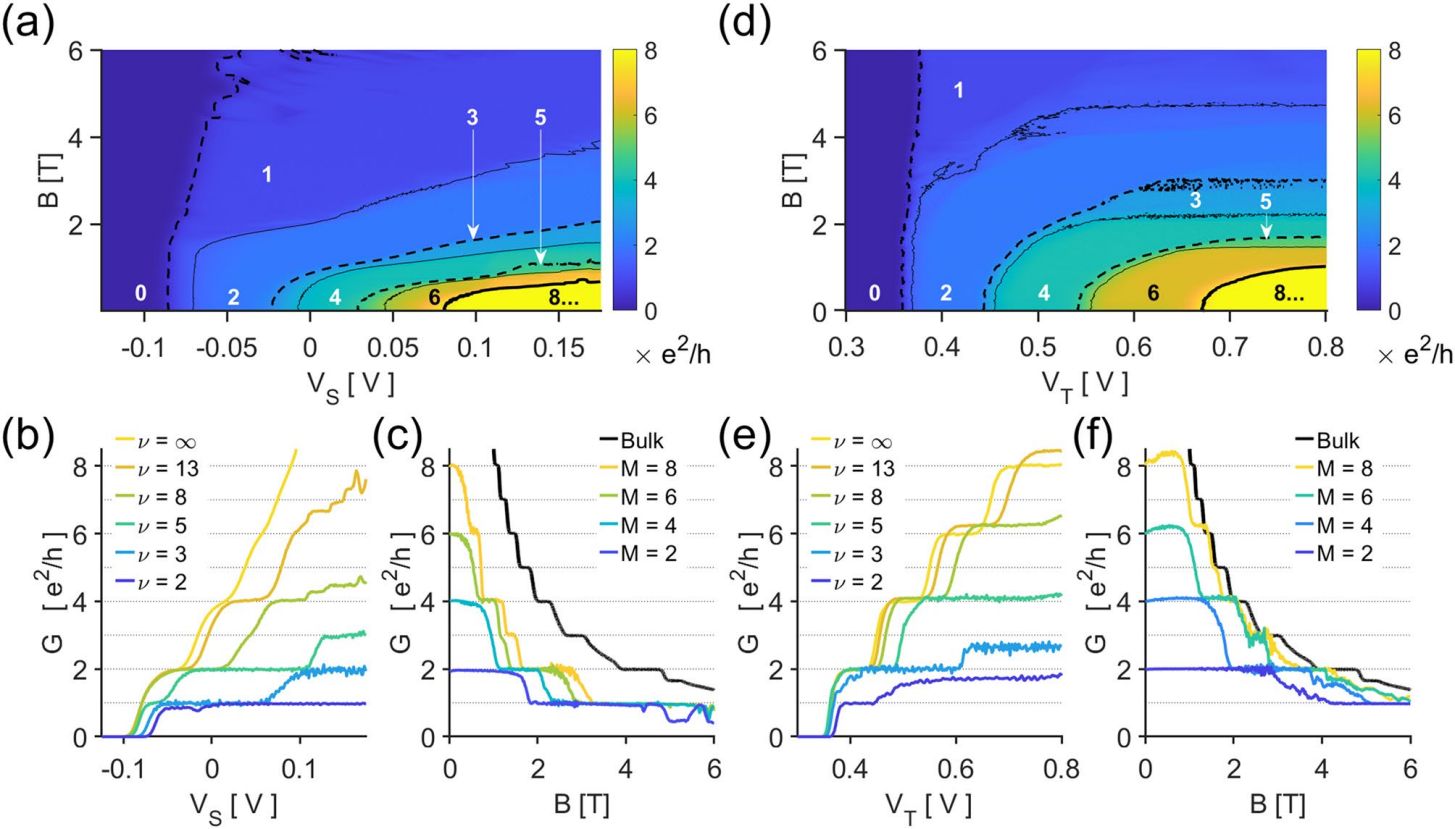
Magnetoconductance of the (**a**) c-QPC and (**d**) t-QPC. Solid curves delineate the contour of ${\it{\text{G}}} = 1.5$, $3.5$, $5.5$, and $7.5$ ${\it{\text{e}}^{2} /{\text{h}}}$; dashed curves, ${\it{\text{G}}} = 0.5$, $2.5$, and $4.5$ ${\it{\text{e}}^{2} /{\text{h}}}$. The numbers indicate the quantity of conducting magnetoelectric subbands at each plateau, ${\it{\text{ M}}}$. (**b**) ${\it{\text{V}}_{{\text{S}}}}$ at which the QPC conductance changes are significantly affected by the bulk filling factor ${\it{\upnu }} = 8.8{\text{ T}}/{\it{\text{B}}}$. (**c**) Increasing ${\it{\text{B}}}$ has an effect similar to lowering ${\it{\text{V}}_{{\text{S}}}}$; note that ${\it{\text{M}}}$ is always significantly smaller than its potentially maximum value ${\it{\upnu }}$ at all times. On the other hand, (**d**) ${\it{\text{V}}_{{\text{T}}}}$ at which the t-QPC conductance changes is not affected as much, the conductance being limited by the upper bound ${\upnu }$. (**e**) At high ${\text{V}}_{{\text{T}}}$, the t-QPC regularly approaches ${\text{M}} \approx {\upnu }$.

The magnetoconductance of a c-QPC is directly affected by its subband spacings, Fig. 4a^31^. By inspecting the c-QPC conductance under a magnetic field $B$ of varying strengths, Fig. 4b, we see that $B$ raises $V_{S}$ at which the conductance changes. This is due to the c-QPCs developing magnetoelectric subbands with energy spacing $\hbar \omega_{0B}$^26^:

4 $$\omega_{0B}^{2} = \omega_{0}^{2} + \left( {\frac{eB}{{m^{*} }}} \right)^{2} .$$

That is, increasing the magnetic field has a similar effect to constricting the c-QPC—Fig. 4c. Note that the number of conducting QPC subbands $M \equiv G \times h/e^{2}$ cannot exceed the number of occupied quantum Hall edge modes or, equivalently, the bulk filling factor $\nu$; indeed, the measured c-QPC never reaches the maximum conductance. In order to transmit all edge states, the c-QPC gap must be much larger than the edge channel separation. However, the c-QPC width cannot be significantly increased in situ beyond the fabricated gate dimensions, which may limit the flexibility or miniaturizability of a device. The t-QPC, Fig. 4d, does not share this limitation as the trench gate can control $M$ without needing to modulate $W$. The t-QPC can have larger subband spacings, so the effect of $B$ on ${\it{\upomega }_{0B}}$ is diminished. Therefore, the gate voltage where the conductance changes are less affected than in c-QPCs, Fig. 4e. Consequently, we see in Fig. 4f that the conductance plateau in $B$ is much wider and the maximum transmission ${\it{\text{M}} = \nu}$ can be achieved for a high enough $V_{T}$. This can also be interpreted as the positively biased trench gate attracting mobile electrons and effectively lowering the local filling factor^32^. Such characteristics can increase the flexibility of a device at high magnetic fields.

#### Robustness at high fields

**Figure 5:**
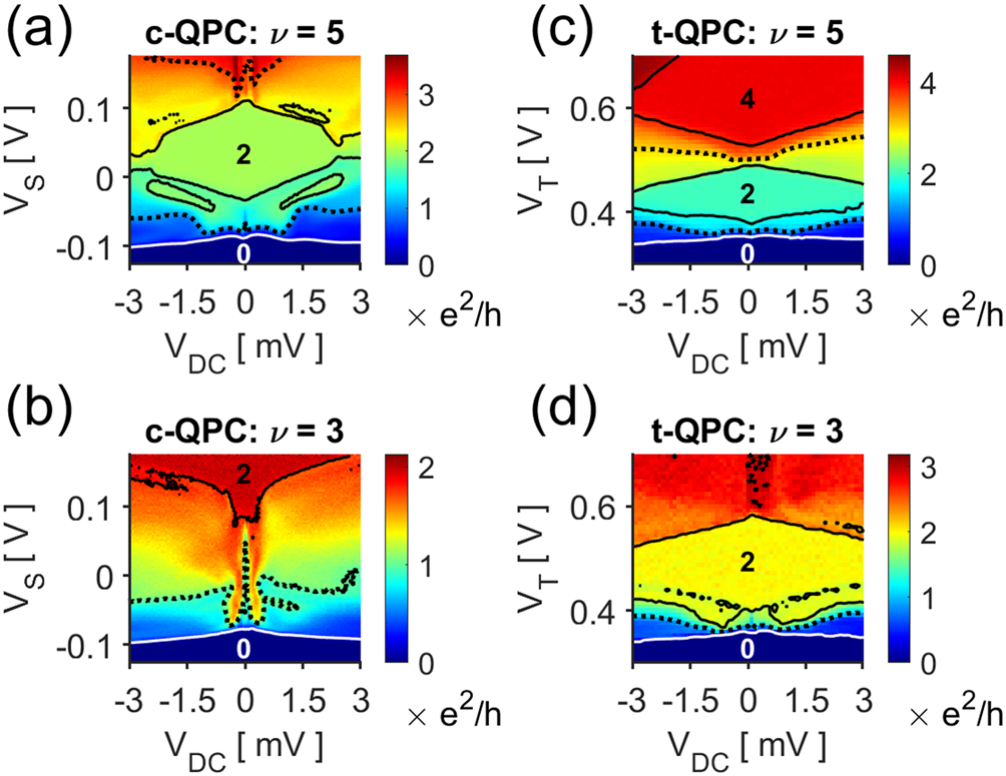
Source-drain biased measurements at various ${\it{\text{B}}}$. The solid white and black curves indicate the contour of ${\it{\text{G}}} = 0.2$, $1.8$, $2.2$ and ${\it{\text{e}}^{2} /{\text{h}}}$; dashed curves, ${\it{\text{G}}} = 1$ and $3$ ${\it{\text{e}}^{2} /{\text{h}}}$. The numbers indicate the conductance at each plateau. (**a**) The c-QPC at (${\it{\text{B}}} = 1.75{\text{ T}}$) ${\it{\upnu }} \approx 5$ presents several peculiarities: the plateau region is not rhombic, islands of ${\it{\text{G}}} = 2{\it{\text{e}}^{2} /{\text{h}}}$ are present, and transition between conductance plateaus are highly nonlinear. (**b**) At (${\it{\text{B}}} = 2.85{\text{ T}}$) ${\it{\upnu }} \approx 3$, few characteristics similar to low-field measurements remain. (**c**) The t-QPC at (${\it{\text{B}}} = 1.80{\text{ T}}$) ${\it{\upnu }} \approx 5$, on the other hand, still closely resembles the result from an ideal QPC. (**d**) At a higher field (${\it{\text{B}}} = 2.70{\text{ T}}$) ${\it{\upnu }} \approx 3$, some irregularities resembling the c-QPC at ${\it{\upnu }} \approx 5$ start to appear.

QPCs at high magnetic fields often find use as non-equilibrium particle injectors^33–35^. However, the device becomes highly sensitive to impurity potentials at the QPC gap and often behaves in uncontrollable ways^36^. Figure 5a,b are c-QPC conductances for varying $V_{DC}$ at bulk filling factors $\nu = 5$ and $3$, respectively. When $\nu = 5$, the previously clear structure seen at low fields becomes obscure, c.f. Figure 3a and 5a. Several aberrant features have appeared as well. From the equal-conductance lines, we see that the conductance plateau is no longer rhombic; islands of $G = 2e^{2} /h$ have appeared, and the conductance transitions have generally become non-monotonic. At even higher fields, $\nu = 3$ in Fig. 5b, the complications intensify, and the measurement can no longer be explained in simple terms. On the other hand, t-QPCs still retain many of its low-field characteristics. For $\nu = 5$, the data qualitatively resembles its equivalent at $B = 0$, c.f. Figures 3c and Fig. 5c; only at higher fields, $\nu = 3$ in Fig. 5d, do the aberrant features such as the conductance islands and obscured transitions start to appear. We attribute the enhanced robustness of a t-QPC to two possible reasons. The trench gate offers greater screening due to the presence of a metallic gate; and the larger potential variation minimizes the relative significance of the impurity potential.

**Figure 6:**
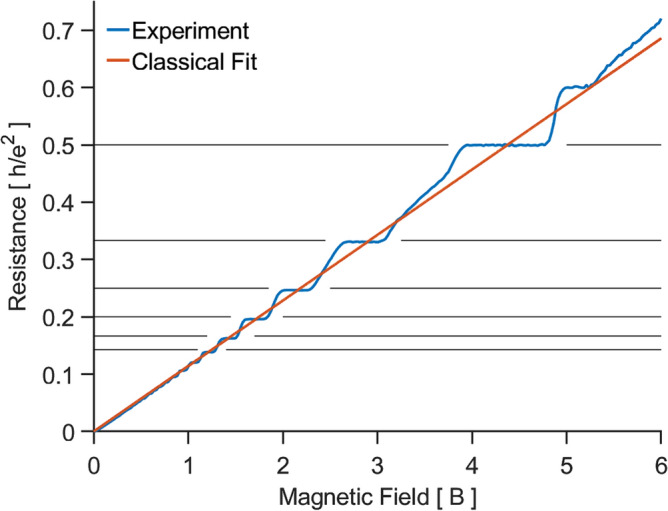
The two-point probe measurement of the wafer’s resistance. The plateaus for various filling factors ${\it{\upnu }} = 2,{ }3,{ } \ldots$ are clearly visible. The classical fit of the 2D sheet metal resistance ${\it{\text{R}} = {\upnu } \times {\text{h}}/{\text{e}}^{2} = {\text{B}}/{\text{B}}_{0} \times {\text{h}}/{\text{e}}^{2}}$ gives us ${\it{\text{B}}_{0}} = 8.8{\text{ T}}$, in agreement with our carrier density measurement.

#### Conclusion

We have analyzed the characteristics of a trench-gated QPC and compared it to those of a conventional QPC on a GaAs/AlGaAs 2DES. Our model and supporting numerical analysis suggest that trench gate modulation leads to larger and more uniform subband spacings over a wider range of conductances, a claim in agreement with our experimental results. Furthermore, the large spacings of the t-QPC were observed and maintained even at high magnetic fields. Specifically, source-drain biased measurements demonstrated that ideal QPC characteristics were better preserved by t-QPCs at filling factors $\nu = 5$ and $3$. Our thorough and multifaceted analysis illustrates the fundamental workings of a QPC, and we believe the t-QPC can be widely applied to future mesoscopic devices for its advantages.

## Methods

### Experiment

The experiment was performed on a GaAs/AlGaAs heterostructure device in a dilution fridge with a base temperature $\approx 100{\text{ mK}}$. The device harbored a two-dimensional electron gas with electron density $n = 2.3 \times 10^{11} {\text{ cm}}^{ - 2}$ and mobility $\mu = 3.8 \times 10^{6} {\text{cm}}^{2} /{\text{Vs}}$ which was controlled using Schottky gates, fabricated using typical electron beam lithography. Placing a voltage on the gates modulates the local 2DES potential; a strong enough negative voltage raises the band minimum above the Fermi level, hence locally depleting the 2DEG and creating an insulating barrier. The unaffected 2DES presented quantum Hall plateaus at high, perpendicular magnetic fields $B$ ^37^. A direct linear fit for the classical Hall effect gave us an estimation for the filling factor $\nu = B_{0} /B$ where $B_{0} = 8.8 {\text{T}}$, Fig. 6. Transport properties of the devices were measured via the usual lock-in technique; a small AC voltage $v_{{{\text{ac}}}} = 10 \mu {\text{Vrms}}$ added upon a DC voltage $V_{{{\text{DC}}}} \le 3.5{\text{ mV}}$ enters the device, and the AC current $I_{{{\text{ac}}}}$ drained from the device is picked up by a lock-in amplifier, Fig. 1a,b. The differential conductance was then calculated by simple division, $G = I_{ac} /v_{ac}$. We also used a homemade transimpedance preamplifier to enhance the signal to noise ratio^38^.

### Simulation and modelling

We calculated the conductance of a simulated device by using KWANT to solve the S-matrix in the tight-binding formalism^39^. A spin-less square crystal of size $81 \times 81$ was defined with hopping parameter $t = \hbar /2m^{*} a^{2}$ and onsite term $U = 4t + \phi$ where $\hbar$ is the reduced Planck constant, $m^{*}$ the effective mass, $a$ the lattice constant, and $\phi$ a spatially varying potential. All units were normalized to realistic experimental conditions:$a = 5 \left( {nm} \right)$ resulting in a $400{\text{ nm}} \times 400{\text{ nm}}$ scattering center; $m^{*} = 0.067 \times m_{e}$ where $m_{e}$ is the bare electron mass, corresponding to the effective mass of electrons in a GaAs/AlGaAs 2DES—$m^{*}$ was set to normalize the all energies, e.g. $t$, in units of ${\text{meV}}$. The reference energy level $E_{f}^{*} = 7 {\text{meV}}$ was used as the Fermi energy.

**Figure 7:**
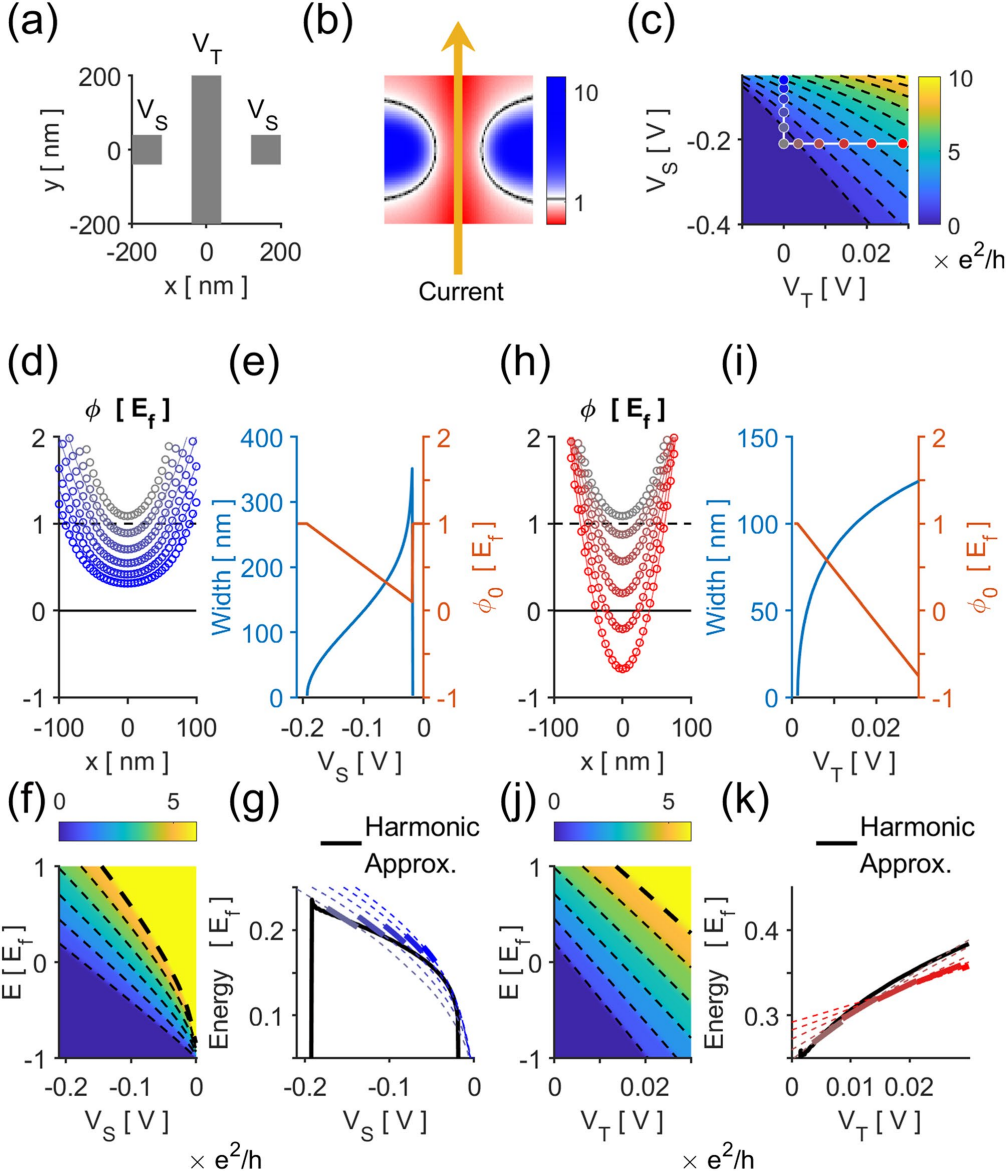
(**a**) Three gates were defined on a $400{\text{ nm}} \times 400{\text{ nm}}$ scattering center for our simulation: a pair of split gates and a trench gate. (**b**) Various voltages ${\it{\text{V}}_{{\text{S}}}}$ and ${\it{\text{V}}_{{\text{T}}}}$ applied to the split and trench gates modulated the onsite potential of the scattering center, through which the conductance was calculated. (**c**) The conductance calculated at the reference Fermi level resembles the experimental results. The c-QPC simulation results correspond to ${\it{\text{V}}_{{\text{T}}}} = 0$, vertical white, and the t-QPC results were inspected at ${\it{\text{V}}_{{\text{S}}}} = - 210{\text{ mV}}$, horizontal white. The lateral potential wells of (**d**) the c-QPC and (**h**) the t-QPC were inspected at said voltage ranges. The corresponding potential width ${\it{\text{W}}}$ and potential minimum ${\it\phi_{0}}$ were extracted from the (**e**) c-QPC and (**i**) t-QPC potentials. The conductance of the (**f**) c-QPC and (j) t-QPC were also calculated at various energy values in order to (**g**,**k**) compare and check for the validity of subband spacings expected by the harmonic approximation using extracted values of ${\it{\text{W}}}$ and ${\it\phi_{0}}$.

Three gates were defined: a pair of split gates and the central trench gate, Fig. 7a. The gates’ electrostatic potential was incorporated by the additional onsite term $\phi$, calculated using the pinned-potential boundary condition^30^, effectively elevated $50{\text{ nm}}$ from the lattice. The split and trench gates were applied a voltage $V_{S}$ and $V_{T}$, respectively, e.g. Figure 7b. The conductance of a current passing through the QPC potential was simulated, Fig. 7c. In the simulation, $V_{T} = 0$ corresponds to the c-QPC.

The potential at $y = 0$, i.e. the QPC at its tightest constriction, were inspected at $V_{T} = 0$ for the c-QPC, Fig. 7d, and $V_{S} = - 210 {\text{mV}}$ for the t-QPC, Fig. 7h. The width $W$ and minima $\phi_{0}$ of the confinement potential were extracted for said voltages, Fig. 7e,i as the parameters used in the analytic toy model, Eq. (2). The modeled and actual subband spacings were compared by calculating the simulated QPC conductance for various energy values, Fig. 7f,j, and tracking the points at which the conductance changed. The spacing between the highest occupied and lowest unoccupied subbands were compared with the model value, Fig. 7g,k, where we see that there is a good agreement between QPC characteristics found by simulation and that estimated by our model.

## Data Availability

The data presented in this study are available upon reasonable request to the corresponding author.
